# Requirement of proline synthesis during Arabidopsis reproductive development

**DOI:** 10.1186/1471-2229-12-191

**Published:** 2012-10-13

**Authors:** Dietmar Funck, Gudrun Winter, Lukas Baumgarten, Giuseppe Forlani

**Affiliations:** 1 Department of Plant Physiology and Biochemistry Biology Section, University of Konstanz, Universitätsstraße 10, 78464, Konstanz, Germany; 2 Department of Life Science and Biotechnology, University of Ferrara, via L. Borsari 46, 44121, Ferrara, , Italy

**Keywords:** Proline metabolism, Gamete and embryo development, Enzyme stability, Subcellular localisation

## Abstract

**Background:**

Gamete and embryo development are crucial for successful reproduction and seed set in plants, which is often the determining factor for crop yield. Proline accumulation was largely viewed as a specific reaction to overcome stress conditions, while recent studies suggested important functions of proline metabolism also in reproductive development. Both the level of free proline and proline metabolism were proposed to influence the transition to flowering, as well as pollen and embryo development.

**Results:**

In this study, we performed a detailed analysis of the contribution of individual proline biosynthetic enzymes to vegetative development and reproductive success in Arabidopsis. In contrast to previous reports, we found that pyrroline-5-carboxylate (P5C) synthetase 2 (P5CS2) is not essential for sexual reproduction although *p5cs2* mutant plants were retarded in vegetative development and displayed reduced fertility under long-day conditions. Single mutant plants devoid of P5CS1 did not show any developmental defects. Simultaneous absence of both P5CS isoforms resulted in pollen sterility, while fertile egg cells could still be produced. Expression of P5C reductase (P5CR) was indispensable for embryo development but surprisingly not needed for pollen or egg cell fertility. The latter observation could be explained by an extreme stability of P5CR activity, which had a half-life time of greater than 3 weeks *in vitro.* Expression of P5CR-GFP under the control of the endogenous P5CR promoter was able to restore growth of homozygous *p5cr* mutant embryos. The analysis of P5CR-GFP-fluorescence *in planta* supported an exclusively cytoplasmatic localisation of P5CR.

**Conclusions:**

Our results demonstrate that potential alternative pathways for proline synthesis or inter-generation transfer of proline are not sufficient to overcome a defect in proline biosynthesis from glutamate during pollen development. Proline biosynthesis through P5CS2 and P5CR is limiting for vegetative and reproductive development in Arabidopsis, whereas disruption of *P5CS1* alone does not affect development of non-stressed plants.

## Background

Reproductive success in plants depends to a large extent on successful adaptation to frequently changing environmental conditions. One of the most prominent adaptive responses to various kinds of stress that result in decreased water availability is the accumulation of the amino acid proline (see [1,2] for recent reviews). However, changes in free proline content also occur during the development of plants growing under non-stress conditions, especially in reproductive organs. Pollen grains, a naturally desiccation tolerant tissue, accumulate large amounts of proline and many flowers secrete proline-rich nectar [3]. Primarily, proline is an essential metabolite for protein synthesis and contributes in a unique way to protein folding, structure and stability. The detailed phenotypic analysis of mutants with defects in proline synthesis can therefore promote our understanding of both primary metabolism and specific functions of proline in plant development.

In plants, glutamate is converted to proline in the cytosol and potentially in plastids by the sequential reactions of pyrroline-5-carboxylate (P5C) synthetase (P5CS) and P5C reductase (P5CR) [1]. In animals and fungi, also arginine or ornithine can serve as precursors for proline, while the separation of the contributing enzymes in different subcellular compartments probably impedes this alternative metabolic pathway in plants [4].

In Arabidopsis, as well as in many other plant species, two P5CS isoforms have been identified with different expression patterns and specific functions in primary metabolism and stress defence [5-7]. *P5CS1* (At2g39800) transcription is strongly induced in response to stress, and *p5cs1* T-DNA insertion mutants were viable but showed reduced basic levels of free proline and a delayed floral transition [6,8]. Additionally, *p5cs1* mutants showed strongly reduced proline accumulation in response to stress, concomitantly with reduced root growth, enhanced production of reactive oxygen species in leaves, and a lower NADP^+^ to NADPH ratio [9,10]. A P5CS1-GFP fusion protein formed cytosolic speckles in embryonic cells, whereas in leaf mesophyll cells of osmotically stressed plants P5CS1-GFP fluorescence was mostly confined in chloroplasts [10].

*P5CS2* (At3g55610) transcripts and P5CS2-GFP fluorescence were mainly detected in meristematic and developing tissues [5,10]. The *P5CS2* promoter was identified as an early target of CONSTANS, a transcriptional activator involved in floral transition [11]. While transcript levels of *P5CS2* did not respond strongly to abiotic stress, they were enhanced in plants undergoing a hypersensitive response to avirulent *Pseudomonas* infection [12,13]. For T-DNA insertion mutants in *P5CS2*, an embryo lethal phenotype has been reported that could be overcome by *ex vivo* cultivation of developing seeds with proline feeding. However, homozygous *p5cs2* mutant plants died before the onset of flowering, thus the specific role of *P5CS2* in reproductive development could so far not be analysed [5,10]. Simultaneous silencing or co-suppression of the two highly similar *P5CS* genes resulted in retarded growth, delayed flowering and reduced apical dominance [8,14].

The second and final step of proline biosynthesis is catalysed by P5CR, which is encoded by a single gene (At5g14800) in Arabidopsis. The SeedGenes project identified T-DNA insertions in *P5CR* as the embryo-defective mutation *emb2772*[15]. Also inhibitor studies on Arabidopsis cell cultures identified P5CR as an essential enzyme for growth [16]. P5CR expression was strongest in young, growing tissues and was induced by stress [17]. Translation efficiency and mRNA stability were identified as additional regulatory steps in P5CR expression [18]. The subcellular localisation of P5CR in plants has so far not been determined unambiguously. While sequence analysis predicts a cytosolic localisation, co-sedimentation of P5CR activity with plastids was observed in pea and soybean [19,20]. Despite the important functions of proline in primary metabolism and in stress tolerance, no experimental data on the localisation of P5CR *in vivo* were available so far.

In this study, we demonstrate that a P5CR-GFP fusion protein is localised exclusively in the cytosol and can fully complement the developmental defects of *p5cr* mutants. Additionally, we report a comprehensive analysis of the requirement of P5CR and the two P5CS isoforms for vegetative and reproductive development in Arabidopsis, while previous reports focused mostly on a single step of proline biosynthesis. In contrast to previous reports, suitable cultivation conditions allowed us the generation of fertile homozygous *p5cs2* mutants and we observed that *p5cs2* but not *p5cs1* mutants showed reduced growth and a delay in the onset of flowering. Infertility of *p5cs1*/*p5cs2* double mutant pollen precluded the generation of double homozygous *p5cs1*/*p5cs2* mutant plants even though double mutant female gametes were fertile. The absence of a functional *P5CR* gene had no influence on the fertility of either male or female gametes, but embryos were aborted after only a few cell divisions in presumably homozygous *p5cr* mutant seeds. Gamete development in the absence of *de-novo* P5CR gene expression could rely upon the extraordinary stability of the P5CR protein.

## Results

### Homozygous *p5cs2* mutants are viable and can produce fertile seeds

Two previous studies have described *p5cs2* T-DNA insertion lines as embryo-lethal or conditionally embryo-lethal and non-fertile. To eliminate potential secondary mutations or ecotype-specific effects, we backcrossed the *p5cs2-1* (GABI452_G01) and *p5cs2-2* (FLAG_139H07) T-DNA insertion lines three and five times, respectively, to the Col-8 accession. In both lines, the segregation pattern was consistent with a single T-DNA insertion and premature abortion of homozygous seeds (data not shown). Homozygous *p5cs2* mutant plants were then generated by *in vitro* cultivation of immature mutant seeds on MS plates containing 60 mM sucrose and 2 mM proline. We observed that also application of mild salt stress to heterozygous *p5cs2-1* mutant plants allowed the formation of approximately 1% homozygous and fertile seeds *in vivo*. Most of the homozygous plants obtained in either way were phenotypically normal, showing only reduced growth compared to wildtype plants ( Additional file 1: Figure S1A). When kept in short-day and low-light conditions, most homozygous *p5cs2* mutant plants completed a normal life cycle and produced viable seeds, which enabled us to analyse development of *p5cs2* mutant plants in direct comparison to wildtype and *p5cs1-4* mutant plants.

### *P5CS2* expression is needed for normal vegetative and reproductive development, whereas *P5CS1* expression seems dispensable

**Figure 1:**
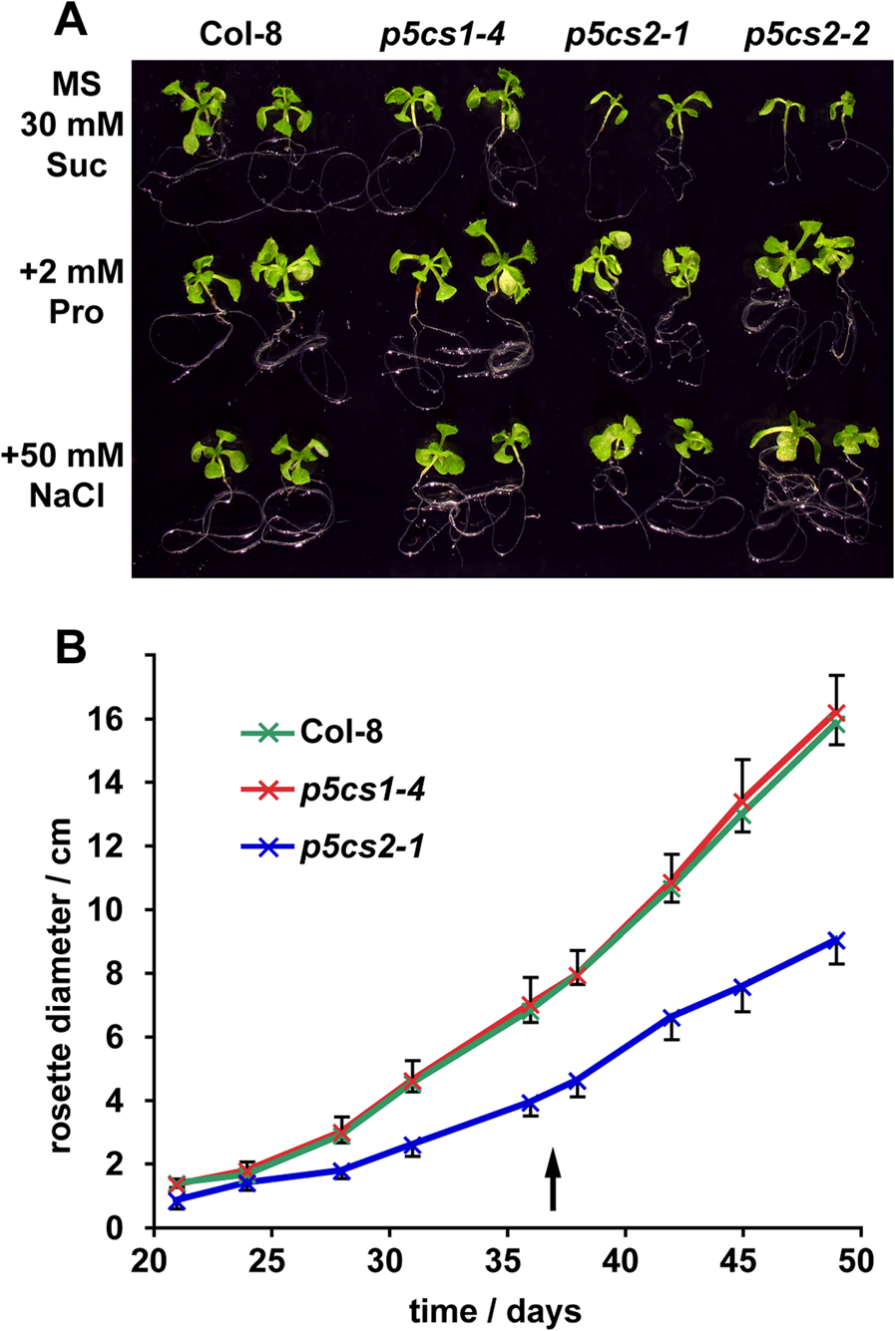
**Retarded growth of *p5cs2 *mutants. A**: Sixteen-day-old seedlings grown in sterile culture under short-day conditions on MS medium with 30 mM sucrose (Suc) and additionally 2 mM proline (Pro) or 50 mM NaCl. **B**: Rosette diameters of plants cultivated in soil under short-day conditions. The arrow indicates the time of fertiliser application, error bars indicate SD of 10≤N≤12 individuals. The size difference between *p5cs2-1 *mutants and the wildtype was significant (p<0.05 by student’s *t*-test) for all timepoints.

When grown in axenic culture, homozygous *p5cs2* seedlings showed a growth-defect that could be complemented by feeding with external proline or application of mild salt stress (Figure 1A). At low mineral or sucrose concentrations, *p5cs2* mutant seedlings frequently failed to establish autotrophic growth ( Additional file 1: Figure S1B). In soil, the rosettes of *p5cs2-1* mutant plants reached only 57% of the size of wildtype plants (Figure 1B), an effect that was even more pronounced when no mineral fertiliser was applied (data not shown).

**Figure 2:**
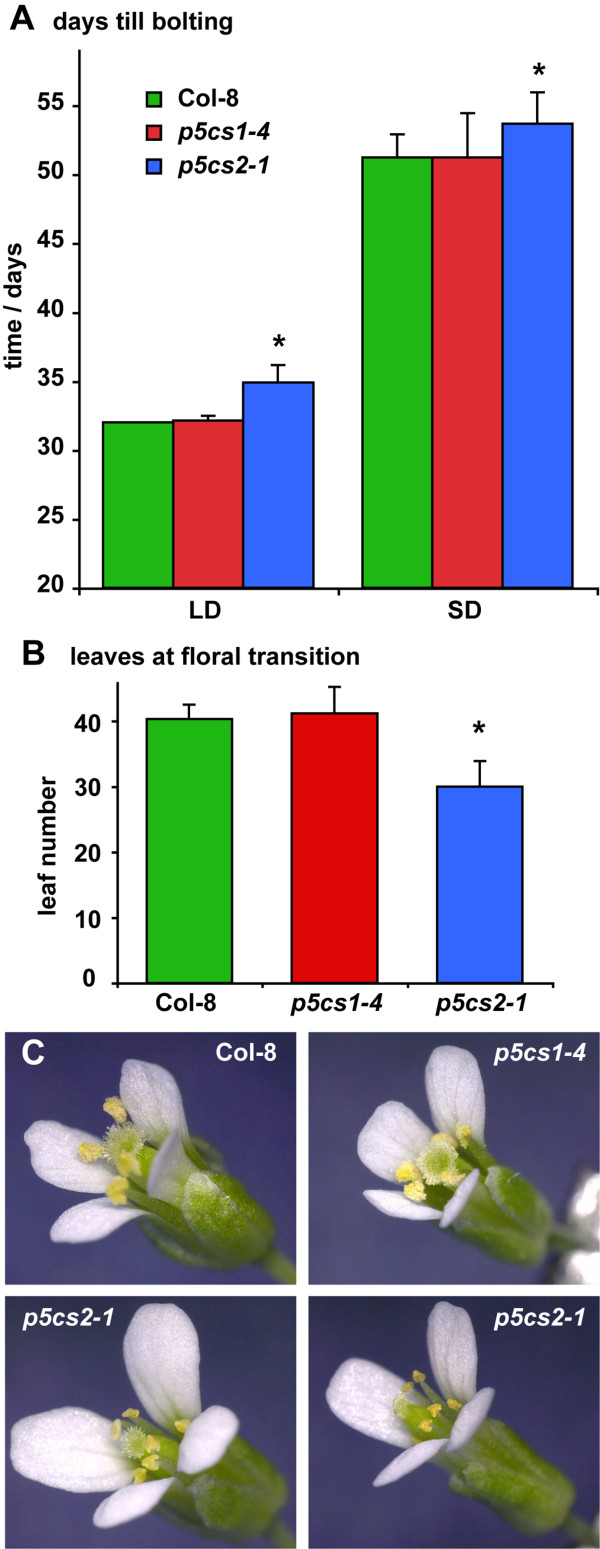
**Defects of *p5cs2-1 *mutants in reproductive development. A**: Time till bolting (bolts longer than 1 cm) in long-day (LD) and short-day (SD) conditions. **B**: Leaf number at the onset of bolting of plants cultivated under short-day conditions in the greenhouse. **C**: Representative flowers of wildtype and *p5cs *mutant plants cultivated under long-day conditions. Error bars in A and B indicate SD of 8≤N≤12 individuals; * indicates significant difference from Col-8 (p<0.05 by students *t*-test).

With respect to floral transition, *p5cs2-1* plants had fewer primary rosette leaves at the onset of bolting, which was delayed by more than 2 days under both short-day and long-day conditions (Figures 2A, B). Occasionally, *p5cs2* mutant plants formed elongated or slightly rolled leaves and such plants mostly failed to produce seeds (data not shown). Cultivation in long-day conditions increased the propensity of *p5cs2* mutant plants to form abnormal leaves and decreased the proportion of fertile plants. In infertile *p5cs2* flowers, the anthers were collapsed and the petals were elongated (Figure 2C). Fertility of mildly affected flowers could be restored by pollination with pollen from a different plant. In *p5cs2* mutants with stronger phenotypic abnormalities, also malformations of the pistil and the stigma were observed, extending to disturbances in floral organ identity and architecture of the whole inflorescence. Such flowers could not be fertilised by cross-pollination (data not shown).

To analyse additionally a potential influence of *P5CS1* expression on growth and development, also the knockout mutant line *p5cs1-4* (Salk_063517) was backcrossed three times to Col-8, and homozygous progeny was included in all experiments. Contrary to the observations of [5], *p5cs1-4* mutant plants did not show any deviations from the wildtype with respect to growth, flowering time, inflorescence architecture or fertility under non-stressful cultivation conditions, irrespective of the light period and the light intensity (Figures 1 and 2).

### *P5CS* expression is required for male fertility

During backcrossing, heterozygous *p5cs2* mutant plants produced less than the expected sulfadiazine (*p5cs2-1*) or BASTA (*p5cs2-2*) resistant progeny (Table 1). Application of mild salt stress increased the mutant allele transmission in both *p5cs2* insertion lines, presumably by induction of *P5CS1* expression. Reciprocal crossing of heterozygous *p5cs2* to wildtype plants indicated that reproductive development is slightly impaired in both the male and the female germlines (Table 1).

**Table 1:** **Transmission of *p5cs2 *mutant alleles by self-pollination and in reciprocal crosses with wildtype plants**

**Genotype pollen donor**^**1**^	**Genotype pollen recipient**^**1**^	**Treatment**	**Offspring with *p5cs2 *allele [% ±SD**^**2**^**]**	**Expected frequency**^**3 **^**[%]**	**Seedlings analysed**
A/a1	A/a1	none	58.9 ±1.3*	66.7	474
A/a1	A/a1	50 mM NaCl	64.1 ±0.5	66.7	457
A/a2	A/a2	none	59.1*	66.7	164
A/a2	A/a2	50 mM NaCl	68.2	66.7	176
A/A	A/a1	none	46.4	50	489
A/A	A/a2	none	40.8*	50	309
A/a1	A/A	none	46.1	50	371
A/a2	A/A	none	47.7	50	461

Transmission of the *p5cs2* mutant alleles was determined by sulfadiazine (*p5cs2-1*) or BASTA (*p5cs2-2*) resistance of the progeny after selfing (first four rows) or reciprocal crossing of heterozygous *p5cs2* mutants with wildtype plants.

^**1**^ A: *P5CS2* wildtype allele; a1: *p5cs2-1* mutant allele; a2: *p5cs2-2* mutant allele.

^2^ SD was calculated from three batches of seeds from individual parent plants.

^3^ The expected frequency takes into account that homozygous *p5cs2*/*p5cs2* seeds are usually non-viable in the presence of wildtype or heterozygous seeds in the same silique (compare Additional file 2 FigureS2).

* indicates significant difference from the expected value (p<0.05 by Χ^2^ test with 1 degree of freedom).

To get a deeper insight in the role of proline synthesis in reproductive development, we also crossed the *p5cs2* mutant lines with the *p5cs1-4* mutant. Surprisingly, the frequency of *p5cs2-1* mutant alleles was much lower than expected among the progeny of *p5cs1-4*/*p5cs2-1* double heterozygous plants (Table 2 and Additional file 2: Figure S2). Transmission of the *p5cs2-1* allele after selfing of heterozygous plants dropped from 65.0% in *P5CS1* wildtype background to 52.3% and 26.7% in *p5cs1-4* heterozygous or homozygous background, respectively. Similar results were obtained with the *p5cs2-2* mutant allele.

**Table 2:** **Transmission of *p5cs2 *mutant alleles in the presence of additional *p5cs1-4 *mutant alleles**

**Genotype pollen donor**^**1**^	**Genotype pollen recipient**^**1**^	**Offspring with *p5cs2 *allele [% ±SD**^**2**^**]**	**Expected frequency**^**3 **^**[%]**	**Seedlings analysed**
A/a1 B/B	A/a1 B/B	65.0 ±4.0	66.7	564
A/a1 B/b	A/a1 B/b	52.3 ±3.4*	66.7	521
A/a1 b/b	A/a1 b/b	26.6 ±3.3*	66.7	545
A/A B/B	A/a1 b/b	43.7 ±5.1	50	332
A/A B/B	A/a2 b/b	50.6 ±8.2	50	212
A/A b/b	A/a1 b/b	44.9 ±3.4	50	382
A/A b/b	A/a2 b/b	44.7 ±2.4	50	232
A/a1 b/b	A/A B/B	0.0 ±0.0*	50	166
A/a2 b/b	A/A B/B	1.0 ±1.7*	50	289
A/a1 b/b	A/A b/b	0.0 ±0.0*	50	88
A/a2 b/b	A/A b/b	0.0 ± 0.0*	50	163

Transmission of the *p5cs2* mutant alleles was determined by sulfadiazine (*p5cs2-1*) or BASTA (*p5cs2-2*) resistance of the progeny after selfing (first three rows) or crossing of various parental genotypes.

^1^ A: *P5CS2* wildtype allele; a1: *p5cs2-1* mutant allele; a2: *p5cs2-2* mutant allele; B: *P5CS1* wildtype allele; b: *p5cs1-4* mutant allele.

^2^ values are the mean ±SD of at least 3 independent plants or crossings.

^3^ the expected frequency takes into account that homozygous *p5cs2*/*p5cs2* seeds are usually non-viable in the presence of wildtype or heterozygous seeds in the same silique (compare Additional file 2: Figure S2).

* indicates significant difference from the expected value (p<0.03 by Χ^2^ test with 1 degree of freedom).

We never obtained homozygous *p5cs2-1* plants that were additionally homozygous or heterozygous for the *p5cs1-4* mutation. On the contrary, homozygous *p5cs1-4* mutants that were additionally heterozygous for either of the *p5cs2* alleles were frequently obtained and showed normal growth and development. Attempts of *in vitro* rescue of retarded embryos from plants homozygous for the *p5cs1-4* allele and heterozygous for the *p5cs2-1* allele never produced double homozygous plants.

To determine whether the transmission of *p5cs2* mutant alleles was specifically impeded in the male or the female germline, we performed reciprocal crossings between plants with multiple *p5cs* mutant alleles. Transmission of the *p5cs2* alleles through the female germline was between 44% and 50% when the mother plant was additionally homozygous for the *p5cs1-4* allele, independently of the *P5CS1* genotype of the pollen donor (Table 2). In sharp contrast, transmission of the *p5cs2-1* allele through the male germline was never observed when the pollen donor plant was homozygous for the *p5cs1-4* mutation. For the *p5cs2-2* allele, we observed just 3 seedlings (among 289 analysed) that appeared to have originated from a *p5cs1-4*/*p5cs2-2* double mutant pollen.

### *p5cr* mutations cause embryonic lethality

**Figure 3:**
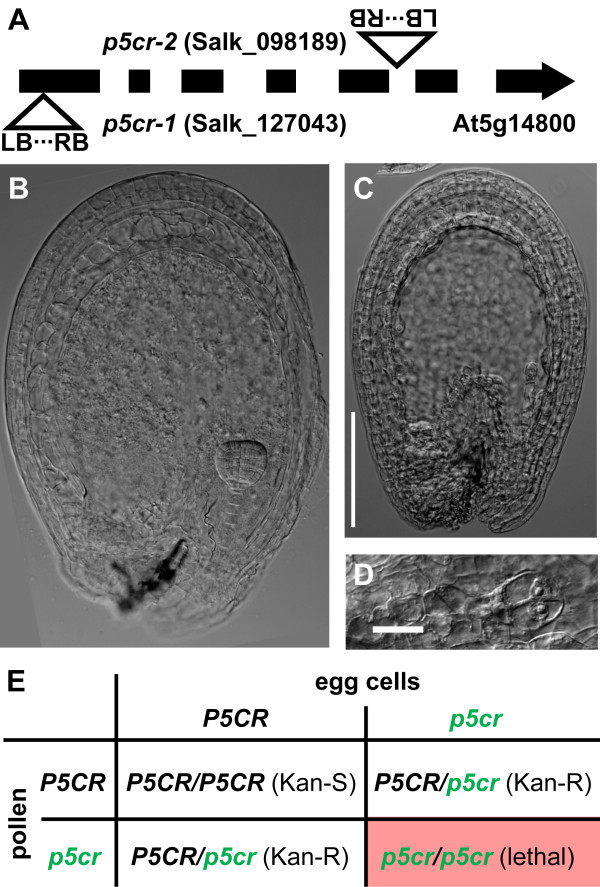
**Embryo abortion in *p5cr *mutants. A**: Graphic representation of the exon-intron structure of the *P5CR *gene and the T-DNA orientations and insertion sites in the *p5cr-1 *and *p5cr-2 *mutant lines. **B**-**D**: Differential interference contrast images of cleared immature seeds. **B**: Normal embryo at late globular stage. **C**: Presumably homozygous *p5cr *mutant seed from the same silique at the same magnification, scale bar = 50 μm. **D**: *p5cr *mutant embryo at the two cell stage at higher magnification, scale bar = 10 μm. **E**: Heredity diagram for self-fertilisation of a heterozygous *p5cr *mutant plant; capital letters indicate wildtype P5CR alleles; minor letters indicate *p5cr *mutant alleles.

The absolute requirement of a functional *P5CS1* or *P5CS2* allele for male fertility prompted us to analyse knockout mutants for the second enzymatic step of proline biosynthesis as well. In Arabidopsis, P5CR is encoded by a single copy gene, and the two T-DNA insertion lines used in our experiments (*p5cr-1* [Salk_127043] and *p5cr2* [Salk_098189]) are listed in the SeedGenes database as embryo-lethal [21]. Although a seed stock from the NASC stock centre (Stock Nr.: N16447) was reportedly derived from a homozygous *p5cr-1* plant, only heterozygous or wildtype plants were obtained from these seeds, indicating that the parent plant was not a homozygous *p5cr* mutant (data not shown). The *p5cr-1* mutant line originally contained at least one additional T-DNA insertion that was eliminated by repeated crossing to the Col-8 wildtype. We determined the exact T-DNA insertion sites in both lines and confirmed that the embryo-lethal phenotype co-segregated with the insertion in the *P5CR* gene (Figure 3A and data not shown). Siliques of plants that were heterozygous for either the *p5cr-1* or the *p5cr-2* allele contained 25% seeds, in which embryo development was arrested at latest after the second division of the embryo proper (Figure 3B-D). The progeny of selfed heterozygous *p5cr* mutant plants segregated 2:1 for kanamycin resistance, as expected forpost-zygotic lethality of homozygous embryos without defects in gametophytic fertility (Figure 3E and Table 3). In reciprocal crossings of heterozygous *p5cr* mutants with the wildtype we observed between 46% and 50% resistant progeny but we could not discriminate whether the deviations from the expected value (50%) were due to either poor expression of the resistance gene or mild defects in gamete development or fertility (Table 3).

**Table 3:** **Transmission of *p5cr *mutant alleles by self-pollination and in reciprocal crosses with wildtype plants**

**Genotype pollen donor**^**1**^	**Genotype pollen recipient**^**1**^	**Offspring with *p5cr *allele [% ±SD**^**2**^**]**	**Expected frequency [%]**	**Seedlings analysed**
D/d1	D/d1	68.5 ±3.6	66.7	773
D/d2	D/d2	68.4 ±1.5	66.7	884
D/D	D/d1	50.0	50	702
D/D	D/d2	45.8*	50	783
D/d1	D/D	48.5	50	581
D/d2	D/D	46.1	50	596

Transmission of the *p5cr* mutant alleles was determined by kanamycin resistance of the progeny after selfing (first two rows) or crossing of various parental genotypes.

^**1**^ D: *P5CR* wildtype allele; d1: *p5cr-1* mutant allele; d2: *p5cr-2* mutant allele.

^2^ SD was calculated from five batches of seeds from individual parent plants.

* indicates significant difference from the expected value (p<0.05 by Χ^2^ test with 1 degree of freedom).

Attempts to rescue putative homozygous *p5cr* mutant embryos by proline feeding *in vitro* failed. Also efforts to promote embryo development of homozygous seeds in siliques of heterozygous parents *in situ* by external proline feeding or induction of internal proline accumulation through salt stress were ineffective. We concluded that despite normal fertility of *p5cr* mutant gametes, *P5CR* expression is essential for embryo development.

### *P5CS* but not *P5CR* expression is essential for pollen development

**Figure 4:**
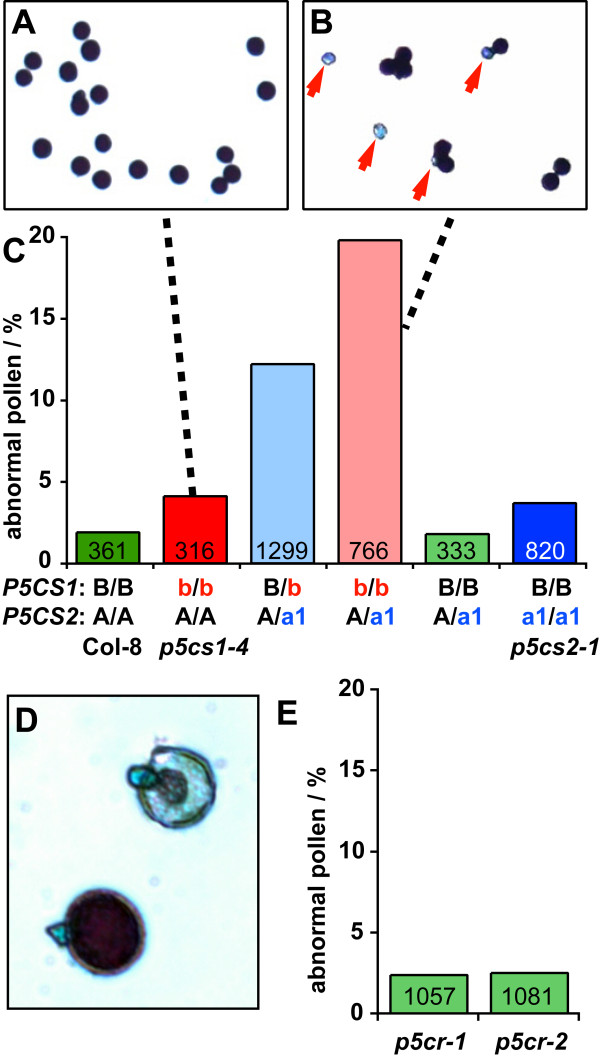
**Defective pollen development in *p5cs *but not *p5cr *mutants . **Pollen was collected from open flowers of 2–4 individual plants and analysed for viability by Alexander staining. The numbers in the columns indicate the total number of pollen grains analysed per genotype. **A**: Pollen of a *p5cs1-4 *single mutant plant, **B**: Pollen of a *p5cs1-4 *mutant plant additionally heterozygous for the *p5cs2-1 *mutation; red arrows indicate non-viable pollen grains. **C**: Abundance of non-viable pollen in various *p5cs *mutant genotypes. Capital letters indicate wildtype alleles, lower case letters indicate mutant alleles (a1 = *p5cs2-1*; b = *p5cs1-4*; Col-8 and homozygous *p5cs *single mutants are additionally labelled for increased clarity). **D**: Normal (lower left) and non-viable pollen grains from a heterozygous *p5cr-1 *plant at higher magnification. E: Frequencies of non-viable pollen grains in heterozygous *p5cr *mutants.

To determine the reason why *p5cr* mutations were transmitted through the male germline, while *p5cs1/p5cs2* double mutations were not, pollen grains collected from open flowers of *p5cs* and *p5cr* mutant plants were stained with Alexander stain (Figure 4). In this procedure, healthy and fertile pollen grains are stained in deep purple, while infertile pollen shows weaker or no staining. We detected slightly less than 2% infertile pollen grains in wildtype and heterozygous *p5cs2-1* plants, and this amount approximately doubled in homozygous *p5cs2-1* or *p5cs1-4* single mutants. Plants heterozygous for mutations in both *P5CS* isoforms produced 12.2% pollen grains that were smaller in size and almost not stained by Alexander stain. The number of non-stained pollen increased to 19.8% in plants homozygous for the *p5cs1-4* mutation and heterozygous for the *p5cs2-1* allele. Similar results were obtained with *p5cs2-2* mutant plants. On the contrary, pollen from heterozygous *p5cr* mutant plants did not show any increase in malformed or unstained pollen, which is in agreement with the previously described normal fertility of *p5cr* mutant pollen (Table 3).

**Figure 5:**
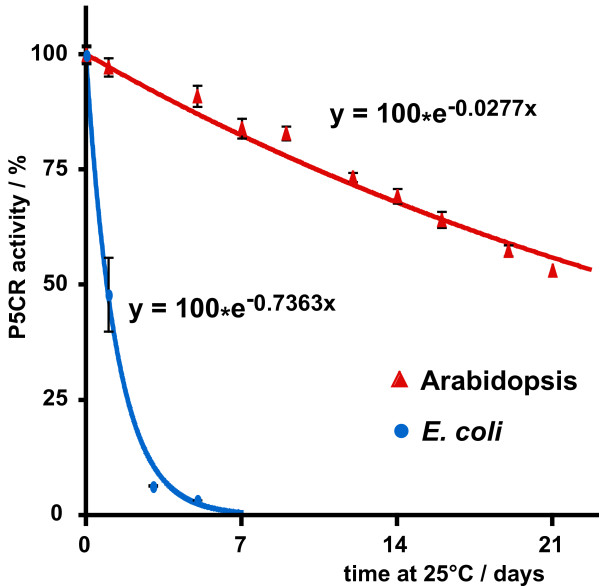
**Stability of Arabidopsis P5CR. **P5CR was partially purified by anion-exchange chromatography from crude extracts of an Arabidopsis cell suspension culture or *E. coli *strain BL21(DE3) (*lon*-, *ompT*-) and sterilised by filtration (0.22 μm pore size). The activity was assayed at increasing time during the subsequent storage at 25 ± 1°C under dim light. Results, expressed as percentage of activity at time 0, are mean ± SD over three independent replicates. The lines represent the exponential function that was generated by least square fitting and was used for calculation of the half-life time of the enzymes.

Pollen fertility in the absence of *P5CR* gene expression suggested an alternative way to ensure proline supply or production in *p5cr* mutant pollen*.* Analysis of publicly available microarray data indicated that only minor amounts of *P5CR* mRNA are accumulated in pollen (data not shown). *De-novo* expression of P5CR protein from an intact gene copy might be dispensable if the P5CR protein present in the pollen mother cell before the separation of the haploid microspores was still active during pollen maturation. To test the likelihood of this hypothesis, the stability of Arabidopsis P5CR was assessed in partially purified preparations from suspension-cultured cells or after heterologous expression in *E. coli* (Figure 5 and data not shown). As a matter of fact, the enzyme was found highly stable upon storage at 25°C, with an estimated half-life of approximately 25 days. In contrast, the endogenous P5CR protein from *E. coli* (encoded by *proC*), isolated and tested under the same experimental conditions, lost more than 50% activity within 24 h. The time from microspore separation to anther opening in Arabidopsis is around 8 days, thus the stability of P5CR *in vivo* could be sufficient to enable the completion of pollen development after meiotic cytokinesis [22].

### Expression of P5CR-GFP complements the *p5cr* T-DNA insertion lines and indicates a purely cytosolic localisation of P5CR

**Figure 6:**
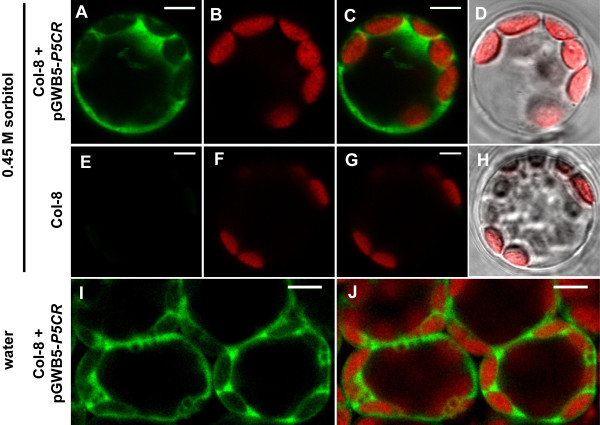
**Subcellular localisation of P5CR. **Confocal images of protoplasts isolated in medium containing 0.45 M sorbitol and a water infiltrated leaf displayed in false colour. **A**-**D**: Protoplast of a stably transformed plant expressing a P5CR-GFP fusion protein under the control of the CaMV-35S promoter. **E**-**H**: Wild type Col-8 protoplast photographed under identical conditions. **I**, **J**: Confocal section through the palisade parenchyma of a water-infiltrated leaf of a P5CR-GFP overexpressing plant viewed from the adaxial side. **A**,**E**,**I**: GFP fluorescence. **B**,**F**: Chlorophyll autofluorescence. **C**,**G**,**J**: Overlay of GFP and chlorophyll fluorescence. **D**,**H**: Merge of chlorophyll fluorescence with a brightfield image. Scale bars = 5 μm.

Surprisingly, crossing of *p5cr* mutants with plants overexpressing a P5CR-GFP fusion protein under control of the Cauliflower Mosaic Virus (CaMV) 35S-promoter did not allow the generation of plants that were homozygous for the *P5CR*-inactivating insertion (data not shown). This failure may be attributable to the poor activity of the CaMV-35S promoter in embryonic tissue ( Additional file 3: Figure S3). Indeed, transformation of heterozygous *p5cr* mutants with a construct containing the native *P5CR* promoter and gene fused to a GFP coding sequence allowed the isolation of plants that were homozygous for the T-DNA insertion inactivating the endogenous *P5CR* gene ( Additional file 3: Figure S3 and Additional file 4: Figure S4). P5CR-GFP expression under control of the native promoter was strongest in young, rapidly growing tissues like young leaves or root tips and was also detected in embryos. Subcellular distribution of P5CR-GFP fluorescence was the same in overexpressing plants and in plants with the native promoter construct, although in the latter case the GFP signal was hardly discernible from autofluorescence signals in mature tissues. Analysis of GFP-fluorescence in isolated protoplasts and in mesophyll cells revealed a uniform cytoplasmatic distribution of P5CR-GFP under both hypoosmotic and hyperosmotic conditions (Figure 6).

## Discussion

A detailed analysis of Arabidopsis mutants in genes involved in proline biosynthesis allowed us to identify the critical steps in development, for which the corresponding enzymes are required. Consistently with previous reports, P5CS1 was not essential for growth and reproduction [5,10]. Moreover, contrary to the data described by Mattioli and colleagues, *p5cs1-4* mutants did not show any delay in the transition to the reproductive phase and no altered morphology of the inflorescence, although the same T-DNA insertion line and similar cultivation conditions were used. Most likely, the original Salk T-DNA insertion line contained additional mutations, which we eliminated by repeated backcrossing.

In this study, we describe for the first time the generation of viable, homozygous *p5cs2* mutant seeds *in vivo*, which enabled us to assess the contribution of P5CS2 to vegetative and reproductive development. In sterile culture, low osmolarity conditions that are suitable to suppress *P5CS1* expression frequently blocked the establishment of autotrophic growth in *p5cs2* mutant seedlings, whereas supply of proline or mild salt stress conditions enabled normal growth. During later development in soil, the inactivation of *P5CS2* by T-DNA insertions caused retarded development but did not prevent successful reproduction when a functional *P5CS1* gene was present. For fertility of homozygous *p5cs2* mutants in soil, the cultivation conditions were critical, as the plants developed generally well in nutrient-rich soil under short-day conditions, whereas they mostly failed to produce viable seeds in long-day conditions. Previous studies reporting non-viability or infertility of homozygous *p5cs2* mutant plants were conducted in areas with a warmer climate and thus support the importance of growth conditions for the viability of *p5cs2* mutant plants [5,10]. Light-dependent regulation of *P5CS1* and *P5CS2* expression was already demonstrated in an earlier study, and both genes were induced by light [12]. Surprisingly, we observed that a longer light period decreased the fertility of *p5cs2* mutant plants, indicating that under these conditions P5CS1 is not sufficient to cover the demand for proline during reproductive development.

Within the flowers, a prominent expression of *P5CS1-GFP* under control of the native promoter was detected in pollen grains, while *P5CS2-GFP* was expressed in the vegetative parts of the anthers [10]. Collapsed anthers at the time of flower opening were the most frequent lesion in flowers of long-day-cultivated *p5cs2* mutant plants, suggesting that *P5CS2* expression in sporophytic anther tissue is pivotal for male fertility under certain growth conditions.

Lethality of homozygous *p5cs2* mutant embryos in siliques of heterozygous parent plants may be explained by slower growth of the homozygous embryos, resulting in a reduced allocation of nutrients and finally leading to premature desiccation and thus death of homozygous embryos when the heterozygous and wildtype siblings have reached maturity. In siliques of homozygous *p5cs2* mutants, all seeds develop at a similar speed and thus desiccation is not induced before the embryos are fully developed. Interestingly, application of salt stress to heterozygous *p5cs2* mutants during flowering allowed survival of at least some homozygous embryos, either by induction or activation of P5CS1 or by a delay in the development of heterozygous and wildtype embryos.

Contrary to *P5CS*, only a single *P5CR* gene is present in Arabidopsis. The early developmental arrest of homozygous *p5cr* mutant embryos suggests a limited transfer of proline from the maternal tissue to the developing embryo. This scenario is likely, since the embryo is fully embedded in endosperm tissue that is equally deficient in *P5CR* expression. Alternatively, continued P5CS activity in P5CR deficient embryos could lead to the accumulation of toxic concentrations of P5C. Toxicity of endogenous P5C accumulation was postulated on the basis of proline-induced toxicity of P5C-dehydrogenase deficient mutants, although a more recent study did not observe toxic effects of externally applied P5C [23-25].

Rescue of homozygous *p5cr* mutants by expression of a P5CR-GFP fusion protein under control of the native *P5CR* promoter confirmed that *P5CR* disruption is the sole reason for the lethality of homozygous *p5cr* mutant embryos. Observation of P5CR-GFP fluorescence exclusively in the cytosol indicates that cytoplasmatic proline synthesis is sufficient to overcome the developmental defect of *p5cr* mutant embryos. Import of P5CS1-GFP and P5CS2-GFP but not P5CR-GFP into plastids of osmotically stressed cells raises new questions about the role of plastids in stress-induced proline accumulation and about the intracellular transport of P5C [10]. Also plant species, for which a chloroplastic localisation of P5CR has been suggested deserve a sincere re-examination with respect to *P5CR* gene copy numbers and P5CR protein localisation [19,20].

Considering the absolute requirement for *P5CR* expression during embryo development, it is puzzling that *p5cr* mutant gametes did not show a clear reduction in fertility. The occurrence of 66% heterozygous offspring after selfing of heterozygous *p5cr* mutant plants might be enabled by a high stability of parental P5CR, either at mRNA or at protein level. While mRNAs of several pollen specific genes were found to be extremely stable in developing pollen, microarray data don’t support the accumulation of *P5CR* transcripts in Arabidopsis pollen [26,27]. However, the activity of Arabidopsis P5CR protein partially purified from suspension cultured cells showed a half-life of almost one month at 25°C. While Arabidopsis P5CR expressed heterologously in *E. coli* had a very similar stability, the enzyme from *E. coli* had a 25-fold lower half-life under the same conditions, despite our efforts to minimise proteolytic degradation by using an *E. coli* strain deficient in the Lon and OmpT proteases. This remarkable stability strengthens the possibility that enough active P5CR protein remains in mutant gametes to complete pollen development after the separation of mutant and wildtype *P5CR* alleles in meiotic cytokinesis, allowing normal rates of fertilisation.

In sharp contrast, *P5CS* expression was essential for pollen fertility, and *p5cs1*/*p5cs2* double mutant ovaries had a reduced success rate to develop fertile seeds even when fertilised by wildtype pollen. Infertility of *p5cs1*/*p5cs2* double mutant pollen impeded the formation of homozygous *p5cs1*/*p5cs2* double mutant embryos ( Additional file 2: Figure S2). These results suggest that parental P5CS mRNA and protein are not stable enough to meet the high demand for proline in pollen and, to a lesser extent, in ovaries. The observation of less than 50% defective pollen grains in flowers of plants with a single functional *P5CS* allele indicates that some double mutant pollen was completely degenerated and no longer recognisable when pollen from open anthers was analysed. Induction of specific transporters for proline in flowers and especially in pollen has been reported, but proline transport is evidently not able to substitute for P5CS-dependent proline synthesis from glutamate in gametes [1]. Lethality of *p5cs1*/*p5cs2* double mutant pollen further implies that no other pathway to produce P5C, *e.g.* from ornithine, is sufficiently active in developing pollen to compensate for the lack of *P5CS* expression.

## Conclusions

With the careful physiological and genetic characterization of insertion mutants in the genes *P5CS1*, *P5CS2* and *P5CR* we demonstrated that both P5CS and P5CR enzyme activities are essential for successful sexual reproduction in Arabidopsis. Obviously neither potential alternative pathways nor cell-to-cell transport of proline can rescue a defect in proline biosynthesis from glutamate. The contrast between sterility in *p5cs1*/*p5cs2* double mutant pollen and normal pollen fertility in the absence of a functional *P5CR* gene can be explained by an unusual stability of the P5CR protein. The observation of an exclusively cytosolic localization of P5CR-GFP raises interesting new questions regarding the intracellular distribution of proline as well as the role of plastids in proline biosynthesis.

## Methods

### Plant material and growth conditions

Arabidopsis (*Arabidopsis thaliana* (L.) Heynh., accession *Col-8*) and T-DNA insertion lines were obtained from the NASC (GABI452_G01, *p5cs2-1*; Salk_063517, *p5cs1-4*; Salk_127043, *p5cr-1*; Salk_098189, *p5cr-2*) or from the INRA Versailles Resource Centre (FLAG_139H07, *p5cs2-2*). Presence of the T-DNA and allelic status were verified by PCR and sequencing of the T-DNA flanking regions. Primer sequences are given in Additional file 5: Table S1. Plants were cultivated axenically as described in [24]. For selection of transgenic plants, the medium was supplemented with 50 μg/ml kanamycin, 5 μg/ml sulfadiazine or 10 μg/ml BASTA (Bayer CropScience, Monheim, Germany). For seed production, plants were kept in a greenhouse with a light period of 9 h (short-day) or 16 h (long-day) with day/night temperatures of 21°C/17°C at photon flux densities between 100 and 200 μmol*m^-2^*s^-1^. To mimic the growth conditions described by [5], plants were kept in an incubator with 16 h of illumination (300 ±30 μmol*m^-2^*s^-1^) at day/night temperatures of 24°C/20°C.

A heterotrophic (white) cell culture was maintained in liquid MS medium supplemented with 3% (w/v) sucrose, 2x MS vitamin mix, 0.5 mg/l 2,4-dichlorophenoxyacetic acid, 0.5 mg/l benzylaminopurine and 0.2% (v/v) Plant Preservative Mixture (Plant Cell Technology, Washington, DC, USA) under constant agitation (100 rpm) in dim light.

### *P5CR-GFP* constructs and imaging

The open reading frame of *P5CR* or a genomic fragment containing 1700 bp of 5’ upstream sequence and the entire *P5CR* gene without the stop codon were amplified by PCR from full-length ORF clone U13409 (ABRC, Columbus, OH, USA) or from genomic Col-8 DNA, respectively. Sequences of PCR primers are given in Additional file 5: Table S1. The resulting PCR fragments were purified and integrated into pENTR by directional TOPO cloning (life technologies, Carlsbad, CA, USA). Subsequently, the inserts were transferred from pENTR to pEarleyGate103 (CD3-685, ABRC), pGWB5 or pGWB4 [28] by LR-recombination (life technologies). Sequencing of the resulting constructs demonstrated in-frame fusion of *P5CR* to the *GFP* gene. Transformation of Arabidopsis plants by floral dip was performed according to [29]. Epifluorescence imaging was performed as described in [24]. For confocal microscopy, protoplasts were isolated from P5CR-GFP expressing transgenic plants and Col-8 according to the method described by [24]. Protoplasts or water-infiltrated leaves were viewed with a 40x oil immersion lens on a Zeiss LSM 510 Meta confocal microscope (Zeiss, Oberkochen, Germany). GFP and chlorophyll fluorescence signals were sequentially captured with the Meta detector of the confocal microscope (GFP: 497–550 nm, chlorophyll: 657–690 nm). Overlay of the images and adjustment to identical brightness and contrast settings was performed in ImageJ and Adobe Photoshop. Whole flowers were photographed with a VHX 500F digital microscope (Keyence, Osaka, Japan).

### Embryo and pollen analysis

For analysis of developmental defects of embryos, immature seeds were isolated under a stereo microscope and cleared in an 8:3:1 (w:v:v) mixture of chloral hydrate:water:glycerol. Images were captured using differential interference contrast (Nomarski) optics and a black-and-white digital camera. Pollen was isolated by vigorously shaking open flowers in ice-cold 300 mM mannitol and centrifugation for 1 min at 12000 g. Viability staining was performed according to [30] for 16 h at 50°C. Images of stained pollen were captured at 100 or 400-fold magnification with a digital colour camera.

### P5CR purification and enzymatic assay

Plant and bacterial cells were homogenized in an ice-cold mortar with 1 g*(g cells)^-1^ quartz sand or 2 g*(g cells)^-1^ alumina, respectively. The homogenate was resuspended in ice-cold extraction buffer (10 mM Tris–HCl pH 7.4, 0.5 mM dithiothreitol, 0.5 mM EDTA), and centrifuged at 4°C for 10 min at 10000 g. Proteins were precipitated from the supernatant by adding solid ammonium sulphate to 70% saturation and then pelleted by centrifugation. The protein pellet was dissolved in a minimal amount of extraction buffer and desalted by passage through a Bio-Gel P6DG column (Bio-Rad, Hercules, CA, USA). The sample was loaded onto a DEAE-Sephacel (GE Healthcare, Little Chalfont, UK) column equilibrated with extraction buffer. Following extensive washing, proteins were eluted with increasing NaCl concentrations in extraction buffer.

The physiological, forward reaction of P5CR was measured by following the P5C-dependent oxidation of NADH. The assay mixture contained 100 mM Tris–HCl buffer pH 7.4, 0.1 mM MgCl_2_, 2 mM DL-P5C, and 0.25 mM NADH in a final volume of 1 ml. A limiting amount of enzyme (0.05-0.25 nkat) was added to the prewarmed mixture, and the decrease in absorbance at 340 nm was determined at 35°C for up to 10 min against blanks from which P5C had been omitted. The activity was determined from the initial linear rate, with the assumption of an extinction coefficient for NADH of 6,220 M^-1^*cm^-1^. DL-P5C was synthesized by the periodate oxidation of δ-allo-hydroxylysine (Sigma-Aldrich, St. Louis, MO, USA), and purified by cation-exchange chromatography on a Dowex AG50 (200–400 mesh) column.

## Competing interests

The authors declare that they have no competing interests.

## Authors' contributions

DF designed the study and performed or supervised the majority of the experiments. GW produced the confocal images, LB performed the analyses of growth and flowering time, GF isolated and assayed the P5CR enzymes. All authors have contributed to the manuscript and approved its final version.

## Supplementary Material

Additional file 1 **Figure S1. **Developmental defects of homozygous *p5cs2 *mutants. A: Five-week-old plants cultivated in short-day conditions. B: Two-week-old seedlings cultivated axenically on half strength MS medium with 30 mM sucrose. Click here for file

Additional file 2 **Figure S2. **Heredity diagram for *p5cs1*/*p5cs2 *double heterozygous mutants. A indicates wildtype *P5CS2 *allele; a indicates *p5cs2 *mutant allele; B indicates wildtype *P5CS1 *allele; b indicates *p5cs1 *mutant allele; green shading indicates herbicide resistance mediated by the T-DNA insertion in *p5cs2 *mutant alleles; pink shading indicates non-viable gamete or embryo; hatched pink shading indicates that the respective progeny will not be observed because of non-viable pollen. Note that the thick black lines delimit the heredity diagram for a heterozygous *p5cs2 *single mutant, while the red lines delimit the progeny of a heterozygous *p5cs2 *mutant that is additionally homozygous for a *p5cs1 *mutation. Click here for file

Additional file 3 **Figure S3. **Expression of P5CR-GFP in embryos. Epifluorescense and brightfield images of isolated embryos displayed in false colour. A-D: Embryo of a homozygous *p5cr-2 *mutant plant complemented with pGWB4-*P5CR*, containing the native *P5C*promoter and gene fused with *GFP*. E-H: Embryo of a Col-8 wildtype plant transformed with pGWB5-*P5CR*, expressing the *P5CR*-cDNA fused to *GFP *under control of the CaMV-35S promoter. I-L: Embryo of a Col-8 wildtype plant. Scale bar = 20 μm. Click here for file

Additional file 4 **Figure S4. **Subcellular localisation of P5CR-GFP and complementation of the *p5cr-1 *mutant. A-D: Epifluorescence images of homozygous *p5cr-1 *mutants expressing a P5CR-GFP fusion protein under control of the native *P5CR *promoter. A-C: Epidermal and spongy parenchyma cells; scale bar = 20 μm. A: GFP fluorescence. B: Overlay of GFP and chlorophyll fluorescence. C: Brightfield image of the same area. D: Comparison of a P5CR-GFP expressing root tip (left) and a non-transgenic root tip, overlay of a brightfield image with GFP fluorescence, scale bar = 50 μm. E: Schematic drawing of the DNA construct in pGWB4 that was used to complement the *p5cr *mutants in comparison to the wildtype *P5CR *gene. Arrows indicate the binding sites of the primers that were used to determine the genotype of the complemented mutants (see panel I). F-H: Epifluorescence images of epidermal and spongy parenchyma cells of a wildtype plant transformed with pEG103-*P5CR*. Note that overexpression with this construct resulted in the formation of cytosolic protein aggregates; scale bar = 20 μm. F: GFP fluorescence. G: Overlay of GFP and chlorophyll fluorescence. H: Brightfield image of the same area. I: PCR-genotyping of *p5cr-1* mutants complemented by transformation with pGWB4-*P5CR*. See panel E for the primer binding sites in the genomic and the transgenic copy of *P5CR*. J: Schematic drawing of the constructs in pEG103 and pGWB5 for overexpression of P5CR-GFP. Click here for file

Additional file 5 **Table S1. **PCR-primers used in this study. Click here for file
