# Supplementary material for: Requirement of proline synthesis during Arabidopsis reproductive development
Source: BMC Plant Biol. 2012 Oct 13;12:191. doi: 10.1186/1471-2229-12-191 (PMC3493334; doi:10.1186/1471-2229-12-191)
Supplement: Additional file 1 — Figure S1. Developmental defects of homozygous p5cs2 mutants. A: Five-week-old plants cultivated in short-day conditions. B: Two-week-old seedlings cultivated axenically on half strength MS medium with 30 mM sucrose. [file 1471-2229-12-191-S1.pdf]

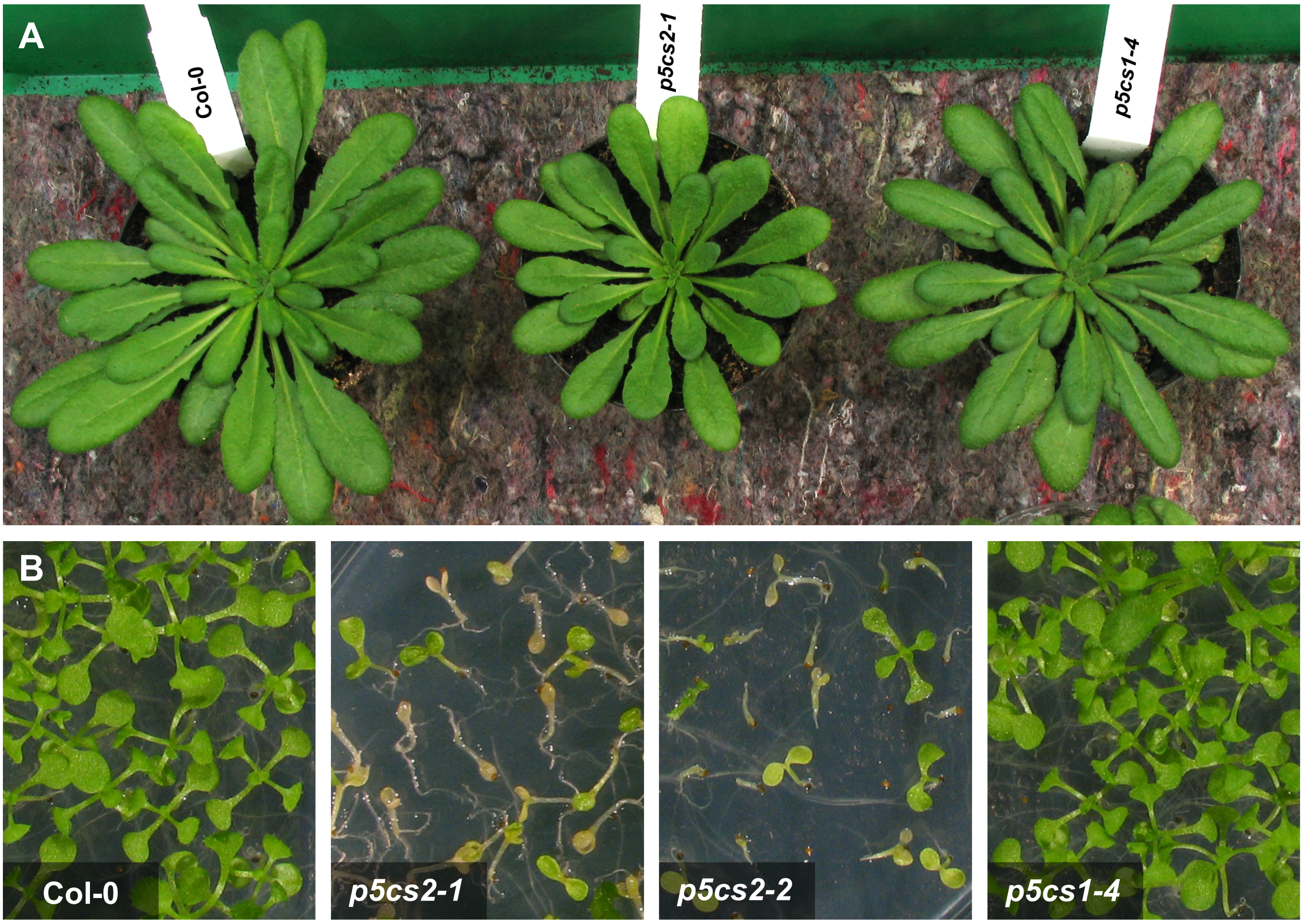

Supplementary Figure S1: Developmental defects of homozygous *p5cs2* mutants
A: Five-week-old plants cultivated in short day conditions. **B**: Two-week-old seedlings cultivated axenically on half-strength MS medium with 30 mM sucrose.
