# Supplementary material for: Requirement of proline synthesis during Arabidopsis reproductive development
Source: BMC Plant Biol. 2012 Oct 13;12:191. doi: 10.1186/1471-2229-12-191 (PMC3493334; doi:10.1186/1471-2229-12-191)
Supplement: Additional file 2 — Figure S2. Heredity diagram for p5cs1/p5cs2 double heterozygous mutants. A indicates wildtype P5CS2 allele; a indicates p5cs2 mutant allele; B indicates wildtype P5CS1 allele; b indicates p5cs1 mutant allele; green shading indicates herbicide resistance mediated by the T-DNA insertion in p5cs2 mutant alleles; pink shading indicates non-viable gamete or embryo; hatched pink shading indicates that the respective progeny will not be observed because of non-viable pollen. Note that the thick black lines delimit the heredity diagram for a heterozygous p5cs2 single mutant, while the red lines delimit the progeny of a heterozygous p5cs2 mutant that is additionally homozygous for a p5cs1 mutation. [file 1471-2229-12-191-S2.pdf]

pollen \ egg cells	A B	a B	A b	a b
A B	AA BB (S)	Aa BB (R)	AA Bb (S)	Aa Bb (R)
a B	Aa BB (R)	aa BB (lethal)	Aa Bb (R)	Aa bb (lethal)
A b	AA Bb (S)	Aa Bb (R)	AA bb (S)	Aa bb (R)
a b (lethal)	Aa Bb (R)	aa Bb (lethal)	Aa bb (R)	aa bb (lethal)

Supplementary figure 2: Heredity diagram for *p5cs1*/*p5cs2* double heterozygous mutants

A indicates wildtype *P5CS2* allele; a indicates *p5cs2* mutant allele; B indicates wildtype *P5CS1* allele; b indicates *p5cs1* mutant allele; green shading indicates herbicide resistance mediated by the T-DNA insertion in *p5cs2* mutant alleles; pink shading indicates non-viable gamete or embryo; hatched pink shading indicates that the respective progeny will not be observed because of non-viable pollen. Note that the thick black lines delimit the heredity diagram for a heterozygous *p5cs2* single mutant, while the red lines delimit the progeny of a heterozygous *p5cs2* mutant that is additionally homozygous for a *p5cs1* mutation.
