# Supplementary material for: Requirement of proline synthesis during Arabidopsis reproductive development
Source: BMC Plant Biol. 2012 Oct 13;12:191. doi: 10.1186/1471-2229-12-191 (PMC3493334; doi:10.1186/1471-2229-12-191)
Supplement: Additional file 3 — Figure S3. Expression of P5CR-GFP in embryos. Epifluorescense and brightfield images of isolated embryos displayed in false colour. A-D: Embryo of a homozygous p5cr-2 mutant plant complemented with pGWB4-P5CR, containing the native P5Cpromoter and gene fused with GFP. E-H: Embryo of a Col-8 wildtype plant transformed with pGWB5-P5CR, expressing the P5CR-cDNA fused to GFP under control of the CaMV-35S promoter. I-L: Embryo of a Col-8 wildtype plant. Scale bar = 20 μm. [file 1471-2229-12-191-S3.pdf]

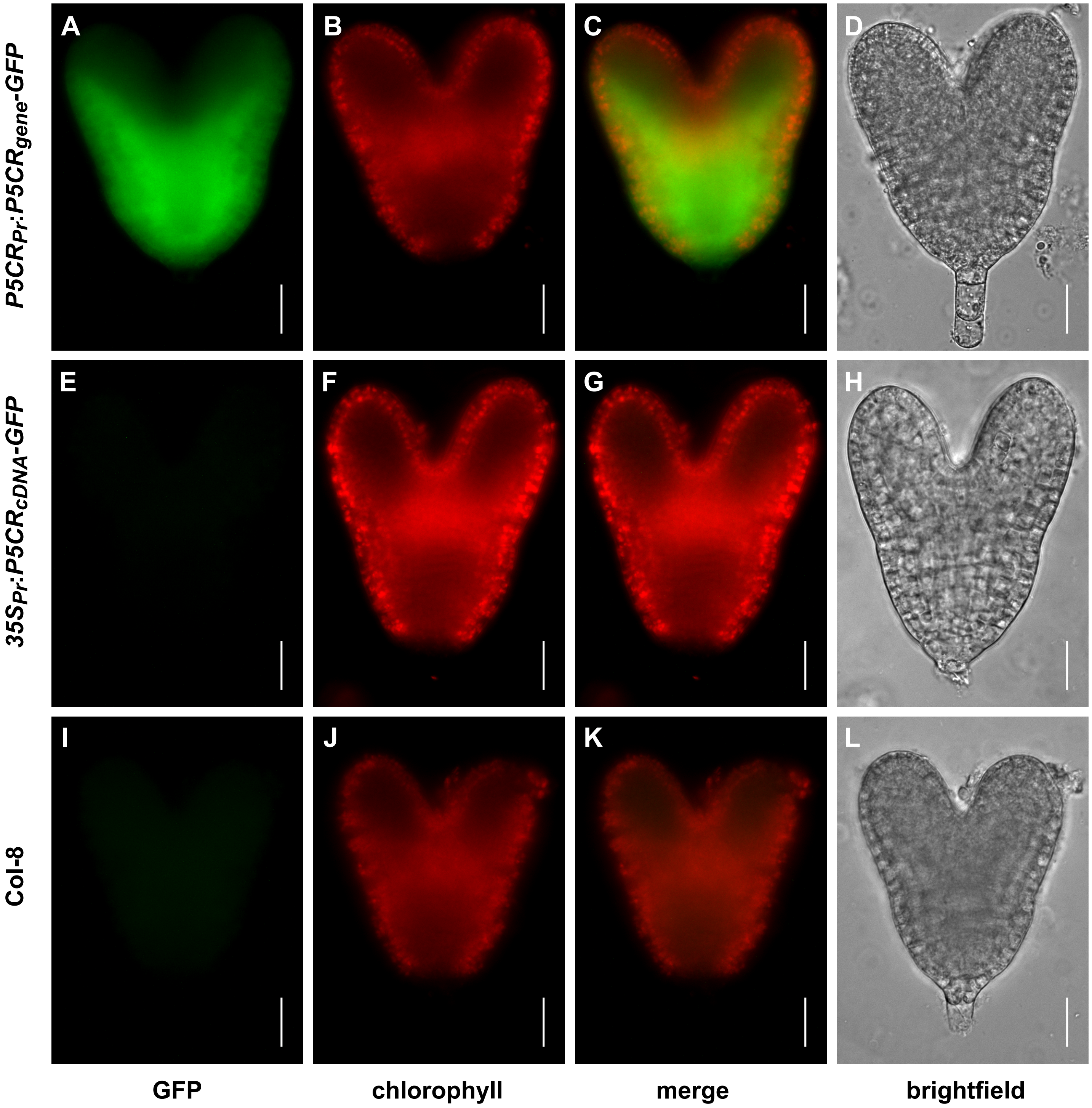

Supplementary figure 3: Expression of P5CR-GFP in embryos

Epifluorescense and brightfield images of isolated embryos displayed in false colour. **A-D**: Embryo of a homozygous *p5cr-2* mutant plant complemented with pGWB4-*P5CR*, containing the native *P5CR* promoter and gene fused with *GFP*. **E-H**: Embryo of a Col-8 wildtype plant transformed with pGWB5-*P5CR*, expressing the *P5CR*-cDNA fused to *GFP* under control of the CaMV-35S promoter. **I-L**: Embryo of a Col-8 wildtype plant. Scale bar = 20 μm
