# Supplementary material for: Requirement of proline synthesis during Arabidopsis reproductive development
Source: BMC Plant Biol. 2012 Oct 13;12:191. doi: 10.1186/1471-2229-12-191 (PMC3493334; doi:10.1186/1471-2229-12-191)
Supplement: Additional file 4 — Figure S4. Subcellular localisation of P5CR-GFP and complementation of the p5cr-1 mutant. A-D: Epifluorescence images of homozygous p5cr-1 mutants expressing a P5CR-GFP fusion protein under control of the native P5CR promoter. A-C: Epidermal and spongy parenchyma cells; scale bar = 20 μm. A: GFP fluorescence. B: Overlay of GFP and chlorophyll fluorescence. C: Brightfield image of the same area. D: Comparison of a P5CR-GFP expressing root tip (left) and a non-transgenic root tip, overlay of a brightfield image with GFP fluorescence, scale bar = 50 μm. E: Schematic drawing of the DNA construct in pGWB4 that was used to complement the p5cr mutants in comparison to the wildtype P5CR gene. Arrows indicate the binding sites of the primers that were used to determine the genotype of the complemented mutants (see panel I). F-H: Epifluorescence images of epidermal and spongy parenchyma cells of a wildtype plant transformed with pEG103-P5CR. Note that overexpression with this construct resulted in the formation of cytosolic protein aggregates; scale bar = 20 μm. F: GFP fluorescence. G: Overlay of GFP and chlorophyll fluorescence. H: Brightfield image of the same area. I: PCR-genotyping of p5cr-1 mutants complemented by transformation with pGWB4-P5CR. See panel E for the primer binding sites in the genomic and the transgenic copy of P5CR. J: Schematic drawing of the constructs in pEG103 and pGWB5 for overexpression of P5CR-GFP. [file 1471-2229-12-191-S4.pdf]

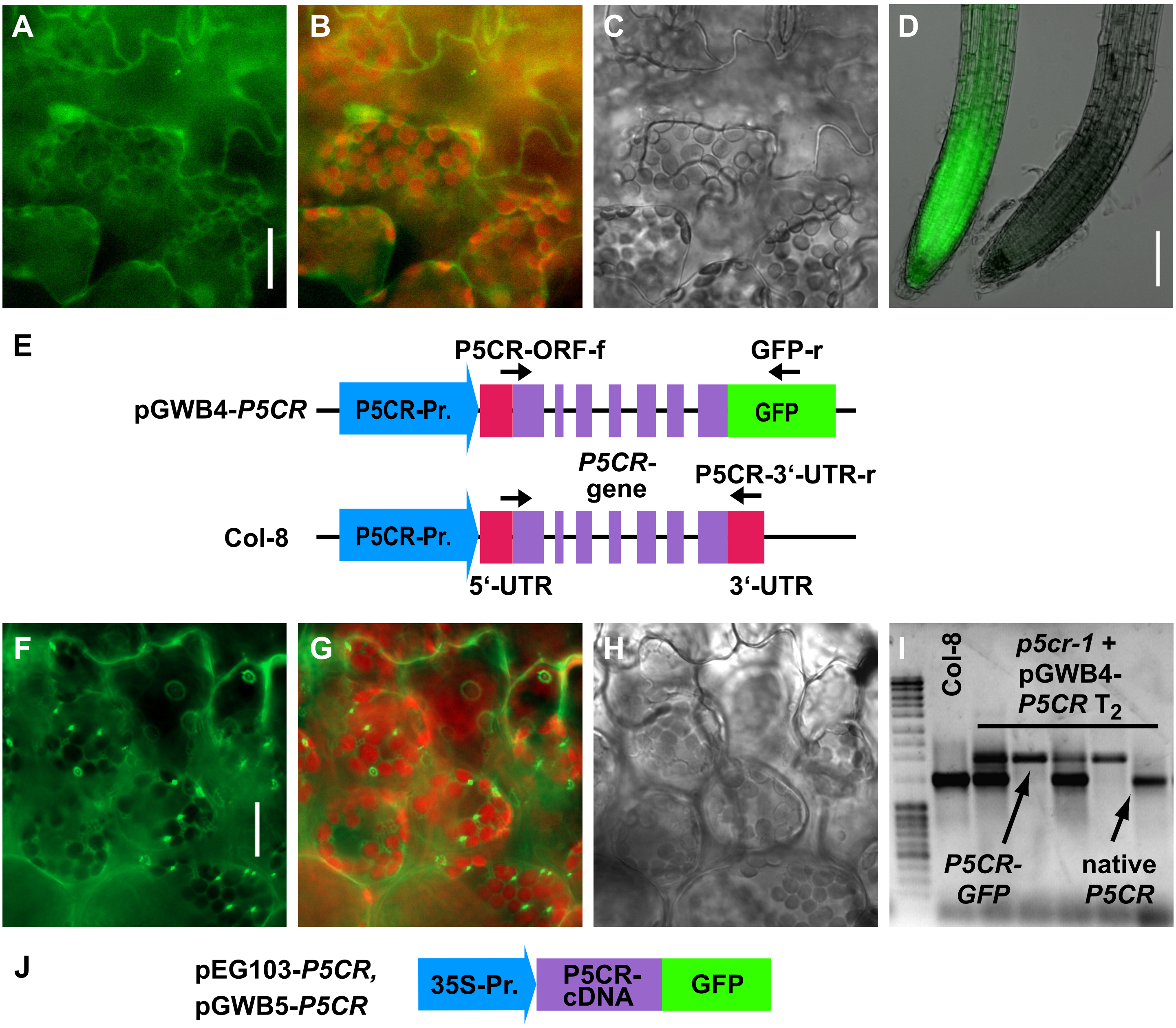

Supplementary figure 4: Subcellular localisation of P5CR-GFP and complementation of the *p5cr-1* mutant

A-D: Epifluorescence images of homozygous *p5cr-1* mutants expressing a P5CR-GFP fusion protein under control of the native *P5CR* promoter. **A-C**: Epidermal and spongy parenchyma cells; scale bar = 20 μm. **A**: GFP fluorescence. **B**: Overlay of GFP and chlorophyll fluorescence. **C**: Brightfield image of the same area. **D**: Comparison of a P5CR-GFP expressing root tip (left) and a non-transgenic root tip, overlay of a brightfield image with GFP fluorescence, scale bar = 50 μm. **E**: Schematic drawing of the DNA construct in pGWB4 that was used to complement the *p5cr* mutants in comparison to the wildtype *P5CR* gene. Arrows indicate the binding sites of the primers that were used to determine the genotype of the complemented mutants (see panel I). **F-H**: Epifluorescence images of epidermal and spongy parenchyma cells of a wildtype plant transformed with pEG103-*P5CR*. Note that overexpression with this construct resulted in the formation of cytosolic protein aggregates; scale bar = 20 μm. **F**: GFP fluorescence. **G**: Overlay of GFP and chlorophyll fluorescence. **H**: Brightfield image of the same area. **I**: PCR-genotyping of *p5cr-1* mutants complemented by transformation with pGWB4-*P5CR*. See panel E for the primer binding sites in the genomic and the transgenic copy of *P5CR*. **J**: Schematic drawing of the constructs in pEG103 and pGWB5 for overexpression of P5CR-GFP.
