# Supplementary material for: Requirement of proline synthesis during Arabidopsis reproductive development
Source: BMC Plant Biol. 2012 Oct 13;12:191. doi: 10.1186/1471-2229-12-191 (PMC3493334; doi:10.1186/1471-2229-12-191)
Supplement: Additional file 5 — Table S1. PCR-primers used in this study. [file 1471-2229-12-191-S5.pdf]

Supplementary table 1: PCR-primers used in this study

Primer:	Sequence:	Used for:
P5CS1-f	CGTAGTGGAAATGGTCTTCTG	genotyping of *p5cs1-4*
P5CS1-r	TATCTCATGAGTTATGCCACTC	
P5CS2-f1	AACCATGGCGGAGATCGATCGTTC	genotyping of *p5cs2-1*
P5CS2-r1	GCAAAACTGAAAATCCGAAGAG	
P5CS2-f2	TGTTTGTTGACCGTTTTCAAAG	genotyping of *p5cs2-2*
P5CS2-r2	GAAGAGAAGCTGCTAAACATTTCAC	
P5CR-f1	ATTAACGACCCTCGTCTCTTC	genotyping of *p5cr-1*
P5CR-r1	TACCAACTTGAGGTTTCACAGAG	
P5CR-f2	GATTCATCAATGTAATTTTGTGTTGAC	genotyping of *p5cr-2*[1]
Salk-LB	TTCGGAACCACCATCAAACAG	T-DNA detection
Salk-RB	TCTGACGTATGTGCTTAGCTC	
Gabi-LB	GGGCTACACTGAATTGGTAGCTC	
Flag-LB4	TGCCAGGTGCCCACGGAATAG	
P5CR-ORF-f	CACCATAATGGAGATTCTTCCGATTCC	cloning of *P5CR* ORF
P5CR-ORF-r	GCTCTGTGAGAGCTCGCGGCTTC	
P5CR-Pr-f	CACCATTAAATCTGAGATGCTCAAGTCTC	Cloning of *P5CR* promoter sequence[1]
P5CR3'UTR-r	TGTGAGGTGAAACAATAGCAG	genotyping of *P5CR-GFP* transgenic plants[2]
GFP-r	TTAGGCCATGATATAGACGTTGTGGCTG	

[1] used in combination with P5CR-ORF-r

[2] used in combination with P5CR-ORF-f
